# Progressive Pulmonary Lesion due to Cystic Fibrosis Transmembrane Conductance Regulator Dysfunction: A Case Study From Japan

**DOI:** 10.7759/cureus.86774

**Published:** 2025-06-25

**Authors:** Kouko Hidaka, Shinichrou Hayashi, Miyuki Nakakuki, Satoru Naruse, Hiroshi Ishiguro

**Affiliations:** 1 Respiratory Medicine, National Hospital Organization Kokura Medical Center, Kitakyushu, JPN; 2 Respiratory Medicine, Kouhoukai Takagi Hospital, Okawa, JPN; 3 Human Nutrition, Nagoya University Graduate School of Medicine, Nagoya, JPN; 4 Gastroenterology, Miyoshi Municipal Hospital, Miyoshi, JPN

**Keywords:** computed tomography, cystic fibrosis, cystic fibrosis transmembrane conductance regulator, lung function, sweat test

## Abstract

Cystic fibrosis (CF) is caused by mutations in the cystic fibrosis transmembrane conductance regulator (*CFTR*) gene, which encodes a chloride ion channel, and occurs frequently in the Caucasian population but rarely in Asia. Elevated sweat chloride using the sweat test is a gold standard for CF diagnosis, but it is not readily available in Japan. A 22-year-old man, who had past histories characteristic of CF, such as recurrent pneumonia, sinusitis, and pneumothorax, was referred to our hospital due to bronchiectasis and bronchial asthma. Examination revealed severely impaired lung dysfunction and abnormal chloride ion concentration in the sweat test corresponding to intermediate values, indicative of CFTR dysfunction. Analysis of his *CFTR* gene failed to detect any CF-causing variants, but showed the haplotype known to express a smaller amount of intact CFTR protein and associated with several pulmonary diseases. A diagnosis was made of bronchiectasis caused by CFTR dysfunction, and he was treated with inhalation solution of dornase alfa, hypertonic saline solution, and tobramycin at the age of 25; however, his lung deteriorated, and he died at the age of 32. As a result of retrospective reviewing of the lung images and functions from childhood, we found that pneumonia in childhood developed to cystic bronchiectasis in adulthood, and obstructive ventilator dysfunction already existed at the age of 13, progressing to the devastating decline of lung function as he grew. Pulmonary disease due to CFTR dysfunction in Japan has a poor prognosis because of challenges to access to the sweat test and a lack of recognition for CF.

## Introduction

Cystic fibrosis (CF) is an autosomal recessive inherited form of bronchiectasis caused by mutations in the cystic fibrosis transmembrane conductance regulator (*CFTR*) gene. CFTR is expressed in the apical membrane of epithelia and operates as a cyclic AMP-regulated chloride ion channel. Loss of function due to *CFTR* variants in both alleles leads to non-functional or less-functional protein products and causes dehydrated luminal fluid in the respiratory and gastrointestinal tract, pancreatic duct, and vas deferens [1]. CF typically shows chronic respiratory tract infections, exocrine pancreatic insufficiency, and male infertility. CFTR mediates chloride ion absorption by the sweat duct, and elevated chloride ion concentrations in sweat show CFTR dysfunction [2].

The chloride ion concentration in sweat is normally 30 mmol or less, and CF is diagnosed if it is 60 mmol or more, coexisting with characteristic symptoms or family history. When the value is in between these ranges, it is defined as intermediate, and other tests of CFTR functions according to the CF guideline are needed [1,2]. However, the sweat chloride test, the gold standard test for the diagnosis of CF [2], is available in a limited number of institutions in Japan, and other tests for CFTR functions are not performed in the country; hence, the diagnosis of CF is often delayed. Standard treatments for CF, such as dornase alfa (a recombinant DNAse) and tobramycin inhalations, were approved in 2012 [3], and the *CFTR* gene variant test is now available in the Japanese Health Insurance system [4]. CF occurs frequently in the Caucasian population but rarely in Asia [5].

We introduce a case of progressive chronic lung disease due to CFTR dysfunction and review lung images and functions from childhood to adulthood in relation to the therapeutic effects of CF standard treatments available in Japan.

## Case presentation

A 22-year-old man was referred to us with bronchiectasis and bronchial asthma, which were diagnosed at age 10 and at age three, respectively. He had a history of recurrent pneumonia in the first three months after birth, bilateral sinusitis at the age of 10 years, and pneumothorax at the age of 15 years, but no problems at birth or development. He had no smoking, drinking, or relevant family history, including consanguineous marriage between parents.

On physical examination, his body mass index (BMI) was 17.0 kg/m^2^ (normal range, 18.5-25.0 kg/m^2^), his respiratory rate was 22/ minute, percutaneous oxygen saturation was 94%, while breathing ambient air, and his breath sounds decreased on both sides of his lung. He was prescribed aerosol inhalation of a mixture of salmeterol xinafoate and fluticasone propionate (Adoair Diskus 250) once a day and one inhalation at a time.

*Pseudomonas aeruginosa*, which was resistant to most antibiotics, except amikacin and levofloxacin, and methicillin-sensitive *Staphylococcus aureus* (MSSA) were repeatedly cultured from his sputum samples. His past history and bronchiectasis, along with respiratory infection by *P. aeruginosa*, were characteristic of CF. His forced expiratory volume in one second (FEV1.0) was 32.1% of the predicted value without reversibility, corresponding to severe obstructive pulmonary dysfunction.

After obtaining informed consent, all exons and their boundaries of the *CFTR *gene were sequenced, and the presence of large deletions was examined by multiplex ligation-dependent probe amplification (MLPA) using the SALSA® MLPA® Probemix P091 CFTR (MRC Holland, Amsterdam, The Netherlands). None of the CF causing variants were detected; however, genotype analysis revealed both alleles carried 12 dinucleotide thymidine guanine repeats (TG) 12/12 at the junction of exon 8 and exon 9, combined with methionine (M) at position 470, which is known to reduce the amount of intact CFTR proteins due to affecting the efficiency of exon 9 splicing (Table 1).

**Table 1:** Analysis of CFTR gene variants *: No change in amino acid sequence; **: Data not provided by ToMMo HGVS: Human Genome Variation Society; dbSNP: Database for Single Nucleotide Polymorphisms; CLINSIG: clinical significance functions; MAF: minor allele frequency; ExAC: Exome Aggregation Consortium; EAS: East Asian population; gnomAD: Genome Aggregation Database; ToMMO: Tohoku Medical Megabank Organization

Variant No.	Gene Name	Feature_ID	Genotype	Annotation	Rank	HGVS.c	HGVS.p	dbSNP	CLINSIG	dbSNP MAF	ExAC_ALL	ExAC_EAS	gnomAD_ALL	gnomAD_EAS	ToMMo	Position	Ref/Alt
1	CFTR	NM_000492.4	Homozygous	Intron_variant	9/26	c.1210-13_1210-12dup	―*	rs3832532	Benign	TG=0.275809	0.1685	0.3974	0.130494	0.50206	―**	chr7:117548606	A/ATG
2	CFTR	NM_000492.4	Homozygous	Missense_variant	11/27	c.1408G>A	p.Val470Met	rs213950	Benign/Likely benign	G=0.417931	0.4829	0.4247	0.556966	0.418956	0.378742	chr7:117559479	G/A

According to the quantitative pilocarpine iontophoresis test (QPIT), the mean sweat chloride ion concentration was 54 mmol/L, which is equivalent to the intermediate value (40-59 mmol/L) of the diagnostic criteria for CF [2]. Since the pancreatic function was sufficient with BT-PABA, this case was classified as probable CF based on the criteria of the Research Committee of Intractable Pancreatic Disease Japan [1], and it was considered that chronic lung damage was due to CFTR dysfunction.

Chest physiotherapy, inhalation of 2.5 mg of dornase alfa (Pulmozyme®) once a day, 300 mg of tobramycin (TOBI®) twice a day, and an inhalation of hypertonic saline solution were initiated. However, he deteriorated due to recurrent hemoptysis from the age of 29, which led to respiratory failure, showing severe hypoxemia with hypercapnia under ventilator support, and he died at the age of 32. Thereafter, we reviewed his lung images, functions, and laboratory data of his entire clinical course.

Persistently low serum sodium ion concentrations (125~137 mEq/L, reference range, 138~146 mEq/L, Table 2), mild inflammatory changes with leukocytosis (9.7×103~18.9×103/μl, reference range 3.3~8.9×103/μl), and hypocholesterolemia (100~109 mg/dl, reference range, 128~219 mg/dl) were observed on his medical records between 17 and 22 years old, but there was no evidence of autoimmune disease or allergic bronchopulmonary aspergillosis. Serum sodium concentrations were reviewed on his medical records between 19 and 29 years of age.

**Table 2:** Data of sodium concentrations in serum samples at each age *The lowest values of serum sodium concentrations at each age; standard values are 138~146 mEq/L.

	19 years	21 years	24 years	26 years	28 years	29 years
Sodium (mEq/L)*	125	128	138	133	133	135

Serial lung function tests were reviewed on his medical records between 13 and 29 years of age (Table 3).

**Table 3:** Serial data of lung function from 13 to 29 years of age FEV1.0: forced expiratory volume in one second; FEV1.0%: forced expiratory volume percent in one second; %FEV1.0: percent predicted forced expiratory volume in one second; FVC: forced vital capacity; %FVC: percent predicted forced vital capacity

		13 years	14 years	19 years	22 years	27 years	28 years	29 years
FEV1.0 (ml)		1610	1580	1830	1500	1360	810	810
FEV1.0% (%)		65.2	61.7	64.4	48.8	51.1	41.4	43
%FEV1.0 (%)		39.9	37.8	43.3	32.1	28.3	17.1	17.2
FVC (ml)		2470	2560	2840	3070	2670	1950	1880
%FVC (%)		91.4	65.3	65.4	65.6	49.2	36.2	35.3

It was estimated that the reduction of FEV1.0% defined with the ratio of FEV1.0 to forced vital capacity (FVC) improved by 10.6% since the beginning of the CF-specific inhalational treatment at the age of 25; however, this must be interpreted with caution as a superficial improvement in the value of FEV1.0% due to the reduction of FVC during this period.

**Figure 1:**
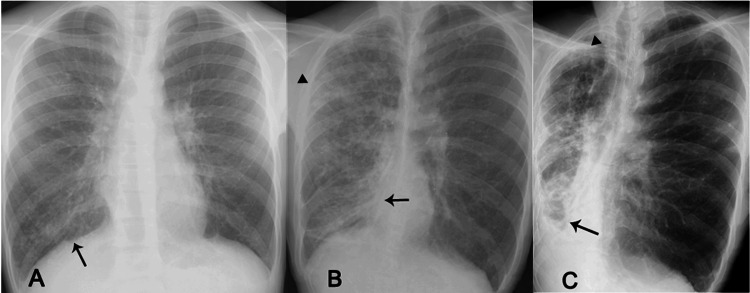
Retrospective findings on serial chest X-rays taken at the age of (A) 11 years, (B) 15 years, and (C) 25 years (A) Mild infiltration in the lower right side of the lung (black arrow); (B) Moderate infiltration in the upper (arrowhead) and volume reduction in the lower regions (black arrow) on the right side of the lung; (C) Cystic change in the apex (arrowhead)and marked bronchiectasis with tracheal shift to the right side in the lower region (black arrow) on the right side of the lung.

**Figure 2:**
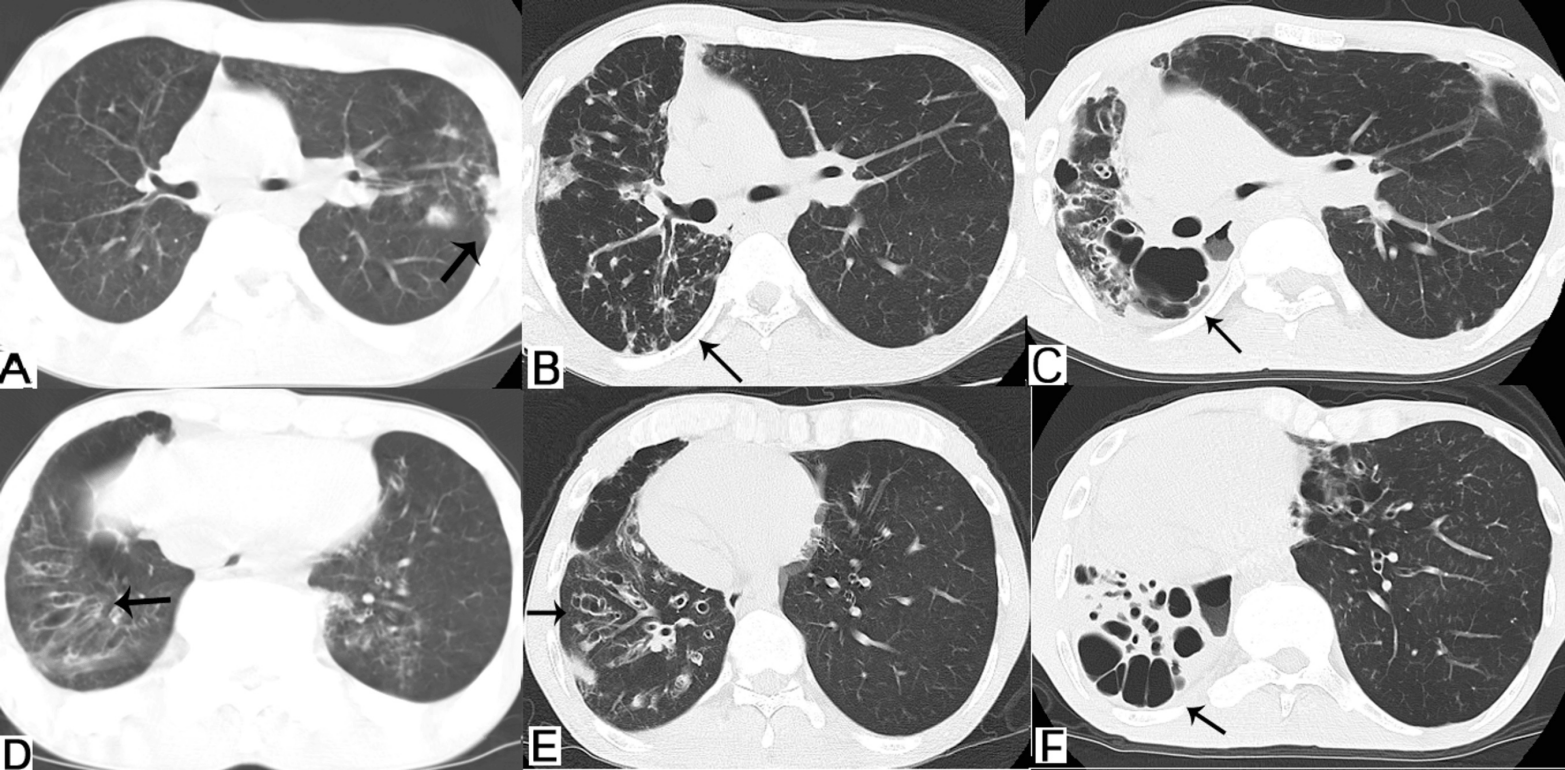
retrospective findings on serial chest CT taken at the age of (A, D) 15 years, (B, E) 22 years, and (C, F) 29 years (A) Mild infiltration (black arrow) in the upper regions on the left side of the lung; (B) Mild bronchiectasis and infiltration (black arrow) in the upper regions on the right side of the lung; (C) Severe bronchiectasis and cyst (black arrow) in the upper regions on the right side of the lung; (D) Cylindrical bronchiectasis (black arrow) in the lower regions on the right sides of the lung; (E) Beaded bronchiectasis (black arrow) in the lower regions on the right side of the lung; (F) Consolidation and multiple cysts (black arrow) in the lower regions on the right side of the lung.

Chest X-ray and CT were reviewed between 11 and 29 years of age (Figures 1, 2). Chest X-rays and CT showed localized bronchopneumonia in childhood, followed by beaded bronchiectasis in adolescents transitioned to cystic bronchiectasis with cicatricial alteration in adulthood. Sinus CT showed fluid retention on both sides of the sinuses, and at the age of 15, developed sinusitis. These sinopulmonary images from childhood to adulthood made it possible to diagnose CF.

## Discussion

Sweat testing and *CFTR *gene analysis are very important for the diagnosis of CF. As for the results of the intermediate sweat test without CF-causing variants in a CF diagnosis, CF Foundation guideline proposed the following insight: the absence of the detection of two CF-causing variants does not exclude a diagnosis of CF; there are individuals with a CF diagnosis in whom two *CFTR* variants have not been detected [2].

Gonska et al. reported a high prevalence of CFTR dysfunction in elderly patients with chronic sinopulmonary disease, highlighting the need for the assessment of CFTR dysfunction using the sweat test as an initial diagnostic test [6]. If QPIT is not available, it may be useful to measure chloride ion concentrations in saliva or in sweat with fingertips [7].

Genetic analysis for the *CFTR* gene in Japanese reported a large deletion spanning exons 16-17b known as a unique variant in Asian races [8], and two major haplotypes of (TG)12-M470 and (TG)11-V470. The former expresses a smaller amount of CFTR protein, and the latter produces proteins with lower intrinsic activity [9]. It was also reported that the haplotypes of (TG)12-M470 show significantly higher frequencies in pulmonary disease patients, such as asthma and bronchiectasis, than in controls [10].

The genotype and phenotype relationship was not fully evaluated in Japanese CF; modifier genes and post-translational modification of CFTR protein are hypothesized as the explanation in patients with positive or intermediate sweat levels but without CF-causing variants [11].

In the reported case, both alleles carrying (TG)12-M470 with no CF-causing variants, along with an undefined factor, might have contributed to lung severity. Infiltration with mild bronchiectasis and subsequent cystic bronchiectasis with cicatricial change may be important and characteristic CF findings on lung images [12], but it was difficult to differentiate them from sinobronchial syndrome, diffuse panbronchiolitis, and non-CF bronchiectasis based on the imaging findings alone.

CF is an extremely rare cause of bronchiectasis in Japan, with an incidence of one in 600,000, in contrast to one in 3,000 in Western countries [1]. Neonatal screening tests for CF, which are popular in Western countries, are not performed in Japan because it is a disease with a low genetic burden. Additionally, limited availability of sweat tests or the lack of recognition for CF might also result in a delay in diagnosis, as in this case, or in undiagnosed patients.

This case showed severe obstructive lung dysfunction at the time of referral to our hospital, and the prognosis may have been better if CF had been diagnosed early with the sweat test.

## Conclusions

This case suggests that the therapeutic intervention for a case of CFTR dysfunction in Asian countries should be performed as early as possible because the prognosis of CFTR pulmonary disease in these regions may be poor. Screening for CFTR dysfunction by sweat test and genetic testing is recommended for referrals to patients with chronic sinopulmonary disease in children and young adults in Asian countries.
